# Nitric oxide-mediated apoptosis of neutrophils through caspase-8 and caspase-3-dependent mechanism

**DOI:** 10.1038/cddis.2016.248

**Published:** 2016-09-01

**Authors:** Megha Dubey, Sheela Nagarkoti, Deepika Awasthi, Abhishek K Singh, Tulika Chandra, J Kumaravelu, Manoj K Barthwal, Madhu Dikshit

**Affiliations:** 1 Division of Pharmacology, CSIR-Central Drug Research Institute, Lucknow, India; 2 Department of Transfusion Medicine, King George's Medical University, Lucknow, India

## Abstract

Neutrophils play an indispensable role in killing of invading pathogens by enhancing reactive oxygen species (ROS) and NO generation, and subsequently undergoing apoptosis. Unlike ROS/NOX2, role of NO/NOS still remains undefined in the apoptosis of neutrophils (PMNs) and the present study attempts to decipher the importance of NO/NOS in the neutrophil apoptosis. Prolonged treatment of human PMNs or mice bone marrow derived neutrophils (BMDN) with NO led to enhanced ROS generation, caspase-8/caspase-3 cleavage, reduced mitochondrial membrane potential and finally cellular apoptosis. NO-induced ROS generation led to caspase-8 deglutathionylation and activation, which subsequently activated mitochondrial death pathway via BID (Bcl-2 family protein) cleavage. NO-mediated augmentation of caspase-8 and BID cleavage was significantly prevented in BMDN from neutrophil cytosolic factor-1 (NCF-1) knockout (KO) mice, implying the involvement of NOX2 in NO-induced apoptosis of PMNs. Furthermore, ROS, NO generation and inducible nitric oxide synthase (iNOS) expression were enhanced in a time-dependent manner in human PMNs and mice BMDN undergoing spontaneous apoptosis. Pharmacological and genetic ablation of iNOS in human PMNs and mice BMDN significantly reduced the levels of apoptosis. Impaired apoptosis of BMDN from iNOS KO mice was due to reduced caspase-8 activity which subsequently prevented caspase-3 and -9 activation. Altogether, our results suggest a crucial role of NO/iNOS in neutrophil apoptosis via enhanced ROS generation and caspase-8 mediated activation of mitochondrial death pathway.

Neutrophils are the most abundant terminally differentiated white blood cells. Although in a normal healthy human, 1–2 × 10^11^ neutrophils are produced daily but hardly a few survive for more than 10 h in circulation.^1, 2^ Neutrophil phagocytose invading pathogens and kill them by producing reactive oxygen intermediates and/or by proteolytic enzymes. Besides pathogen clearance, neutrophils are also detrimental in a number of inflammatory diseases.^3^ Spontaneous apoptosis is thus crucial for neutrophil homeostasis and resolution of inflammation. Neutrophil apoptosis is controlled by apoptotic and survival pathways, which are modulated by pro- and anti-inflammatory cytokines, caspases and calpains. Moreover, a critical balance between reactive oxygen species (ROS) and anti-oxidants is required for cell survival. In neutrophils, ROS is largely produced by the enzyme NADPH oxidase (NOX) which adversely affects their survival.^4, 5, 6^ Yan *et al.*^7^ have recently demonstrated that NOX4 derived ROS following TGF-*β* stimulation induced apoptosis in endothelial cells.

Nitric oxide (NO), a gaseous signalling molecule synthesized by NO synthase (NOS) from l-arginine, regulates several cellular functions such as vasodilation, migration, proliferation, differentiation and apoptosis. Cell death is induced following enhanced levels of NO from inducible nitric oxide synthase (iNOS) during inflammation, ischaemia/reperfusion or by NO donors such as DETA-NO, sodium nitroprusside and S-nitroso-N-acetyl-penicillamine.^8, 9, 10^ Our previous work has demonstrated a dose-dependent pro- and anti-apoptotic effect of NO on promyelocytic cell line HL-60.^11^ Two isoforms of NOS-iNOS and nNOS are constitutively expressed in human and mice PMNs^12^ but their regulation and interplay in neutrophil apoptosis is still enigmatic.

Caspases having a crucial role in the modulation of apoptosis and apoptotic pathways have two components; caspase-8, an initiator caspase^13^ which mediates Fas induced death pathway, and caspase-9, which is vital for the mitochondrial mediated death. Opening of the mitochondrial membrane transition pore leads to cytochrome *c* release into the cytosol-forming apoptosis protease activating factor-1 (Apaf-1), a multimeric complex known as apoptosome which then activate pro-caspase-9. On the other hand, caspase-8 cleaves BID to tBID which translocate to mitochondria and release cytochrome *c*.^5^ Caspase-3, the effector caspase, is important for both extrinsic and intrinsic pathway with well documented role in the regulation of neutrophil apoptosis.^14^ It was shown that the anti-apoptotic effect of NO was related to the inhibition of caspase-3 activation through cGMP-dependent and independent mechanisms.^15^ S-glutathionylation is a redox-based regulatory mechanism which regulates caspase cleavage and its activation. Caspase-3 undergoes glutathionylation at Cys (163, 184 and 220) which prevents its cleavage and activation.^16^ In endothelial cells, TNF-*α* induced caspase-3 cleavage and apoptosis are regulated by caspase-3 glutathionylation/deglutathionylation cycles.^17^

The present study demonstrates the crucial role of NO/iNOS in neutrophil survival. NO-induced ROS generation in human PMNs and mice bone marrow derived neutrophils (BMDN) led to caspase-8 cleavage, activation of BID and initiation of the mitochondrial death pathway. Augmented ROS production and apoptosis in NO pre-treated cells were attenuated in neutrophil cytosolic factor-1 (NCF-1) knockout (KO) mice BMDN or VAS-2870 treated human PMNs suggesting role of NOX in NO mediated initiation of apoptosis. NO-induced deglutathionylation of caspase-3 and -8 suggest redox mediated modulation of neutrophil apoptosis. Moreover, spontaneous apoptosis of BMDN was reduced in iNOS KO mice, iNOS silenced or iNOS inhibitor treated human PMNs, implying the importance of iNOS in neutrophil apoptosis. Altogether, these findings demonstrate the role of caspase-3, -8 and -9 in NO/iNOS induced neutrophil apoptosis.

## Results

### Effect of exogenous NO on the apoptosis of human neutrophils

**Figure 1:**
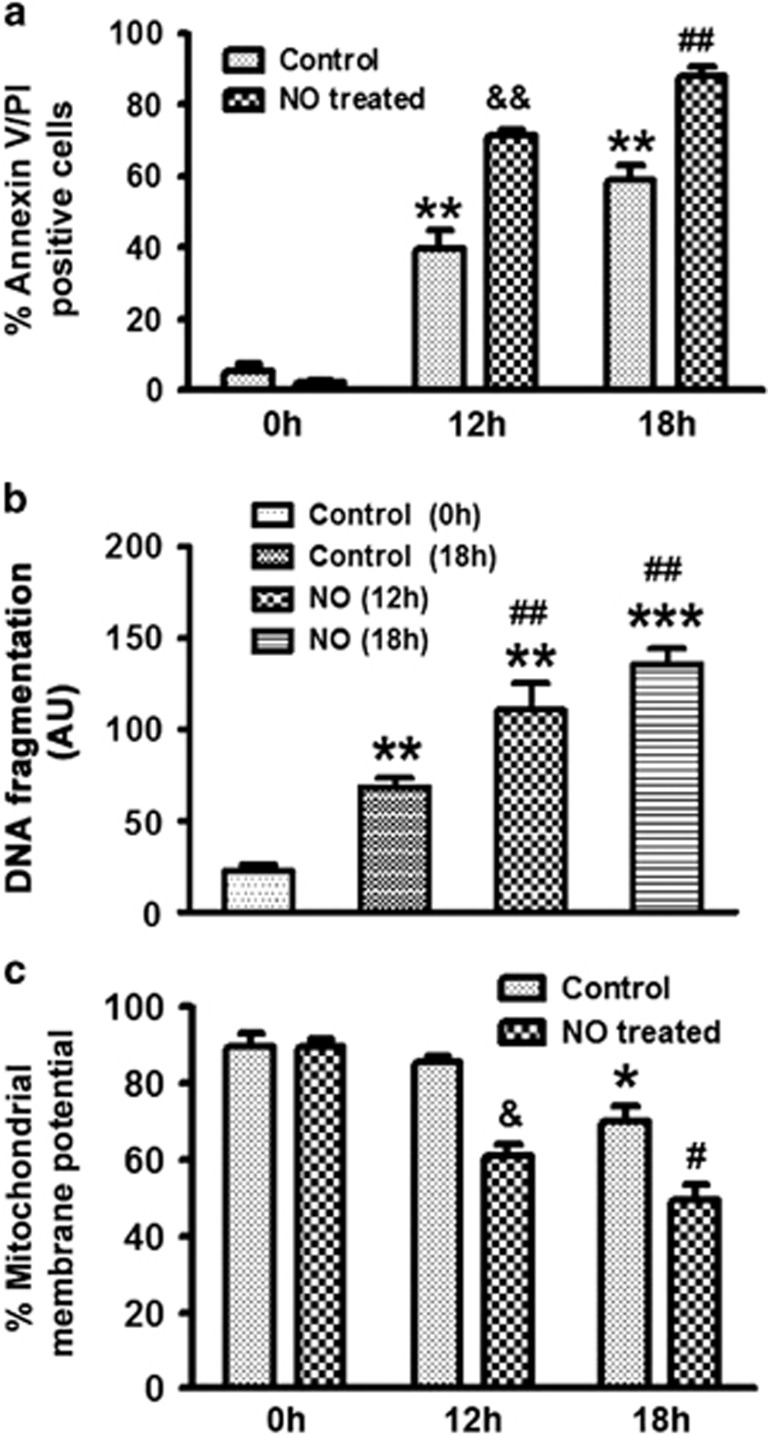
Neutrophil apoptosis in NO and vehicle-treated cells. (**a**) Human PMNs (1 × 10^6^cells/ml) were treated with DETA-NO (300 *μ*M) or vehicle and cultured overnight. Cell apoptosis was monitored by Annexin V-PI labelling (*N*=5). (**b**) Pro-apoptotic effect of NO on DNA damage as assessed by TUNEL assay (*N*=3). Apoptotic cells (control 18 h) indicate healthy vehicle-treated control neutrophils cultured for 18 h. (**c**) Measurement of mitochondrial membrane potential was performed by labelling with JC-1 (*N*=5). Data are shown as the mean±S.E.M. of three to five independent experiments.*P<0.05, **P<0.01, ***P<0.001 *versus* control neutrophils, ^&^*P*<0.05, ^&&^*P*<0.01 *versus* 12 h cultured neutrophils, ^#^*P*<0.05, ^##^*P*<0.01 *versus* 18 h cultured neutrophils

Neutrophils are short lived cells and undergo spontaneous apoptosis during *in vitro* culture which was monitored in a time-dependent manner in presence of DETA-NO. Although at initial time points cell death was not significantly affected, enhanced apoptosis was observed after 12 and 18 h of DETA-NO (300 *μ*M) treatment (Figure 1a; Supplementary Figure S1A) and was additionally confirmed by TUNEL assay (Figure 1b). Mitochondrial membrane potential was found to be reduced to 0.7-fold in presence of NO as measured using JC-1 (Figure 1c).

### NOX-mediated activation of caspase-8 in NO-treated cells

**Figure 2:**
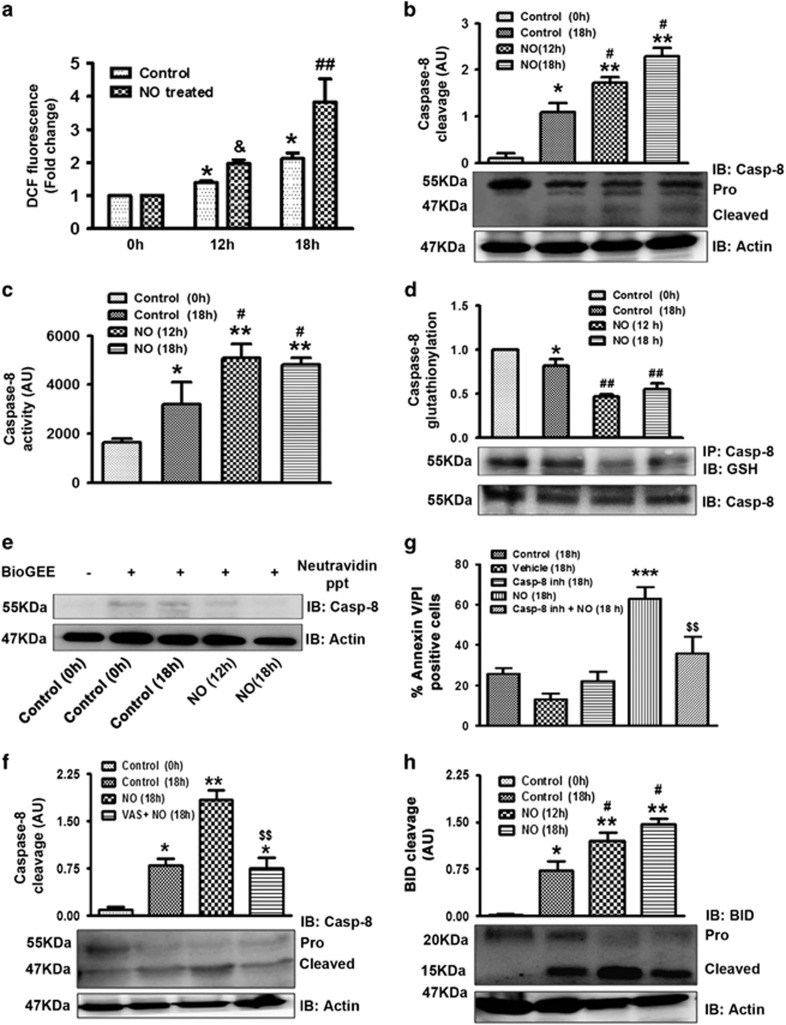
NOX mediated activation of extrinsic death pathway. (**a**) ROS generation as measured by DCF (10 *μ*M). Control and NO-treated human PMNs (1 × 10^6^ cells/ml) were incubated with DCF after 12 and 18 h (*N*=7). (**b**) Caspase-8 cleavage was monitored by Western blotting (*N*=5). (**c**) Caspase-8 activity was assessed in control and NO-treated human PMNs after overnight culture using caspase-8 specific fluorogenic substrate. Fluorescence of the cleaved product was measured using fluorimeter at excitation wavelength 380 nm and emission wavelength 460 nm (*N*=5). (**d**) Caspase-8 glutathionylation level was measured. Caspase-8 was immunoprecipitated from NO-treated neutrophil lysate by anti-pro-caspase-8 antibody and blotted with anti-GSH antibody (*N*=3). (**e**) Detection of caspase-8 glutathionylation by BioGEE labelling (*N*=3). (**f**) Caspase-8 cleavage in VAS-2870 pre-treated human PMNs. Cells (1 × 10^6^cells/ml) were treated with VAS-2870 followed by treatment with NO and *in vitro* culture for 18 h. Caspase-8 cleavage was monitored by western blotting (*N*=3). (**g**) Cell apoptosis was monitored in caspase-8 inhibitor (Casp-8 inh) pre-treated cells. Human PMNs (5 × 10^6^ cells/ml) were incubated with caspase-8 inhibitor (100 *μ*M) followed by treatment with DETA-NO (300 *μ*M). Cells were incubated overnight (18 h) and apoptosis was monitored by Annexin V-PI labelling (*N*=5). (**h**) BID truncation to tBID was assessed by western blotting (*N*=3). Data are shown as the mean±S.E.M. of three to seven independent experiments. *P<0.05, **P<0.01, ***P<0.001 *versus* control neutrophils, ^&^*P*<0.05 *versus* 12 h incubated apoptotic neutrophils, ^#^*P*<0.05, ^##^*P*<0.01 *versus* 18 h incubated apoptotic neutrophils, ^$$^*P*<0.01 *versus* NO-treated neutrophils

We found enhanced ROS generation in overnight incubated cells pre-treated with NO as compared with the control vehicle-treated cells (Figure 2a). Caspase-8 cleavage (Figure 2b) and its activity (Figure 2c) were also enhanced in presence of NO. As caspase activity and cleavage are regulated by glutathionylation, we also studied caspase-8 glutathionylation in control and NO-treated human PMNs. Interestingly, in NO-treated cells we detected deglutathionylation of caspase-8, which might be regulating caspase-8 activity in these cells (Figure 2d). This finding was further confirmed by BioGEE labelling of the neutrophils in presence of NO. Glutathionylated proteins were precipitated with neutravidin beads and caspase-8 was identified by western blot. Caspase-8 glutathionylation was observed to be significantly reduced in NO-treated cells (Figure 2e). Human PMNs treated with NOX inhibitor VAS-2870 (25 *μ*M) for 15 min before the addition of NO, demonstrated reduced apoptosis suggesting role of NOX in NO-induced neutrophil apoptosis (Supplementary Figure S1B). NO-induced capsase-8 cleavage was attenuated in presence of VAS-2870 demonstrating the importance of NOX in NO-induced caspase-8 cleavage (Figure 2f). Moreover, apoptosis was reduced in caspase-8 inhibitor pre-treated cells (Figure 2g). These findings thus demonstrate the involvement of NOX-mediated ROS generation in caspase-8 activation in NO-induced neutrophil apoptosis. Active caspase-8 initiate cleavage of BID which then translocate to the mitochondria. We, therefore, assessed the cleavage of BID in NO-treated human PMNs where it was found to be enhanced (Figure 2h).

### Involvement of mitochondrial death pathway

**Figure 3:**
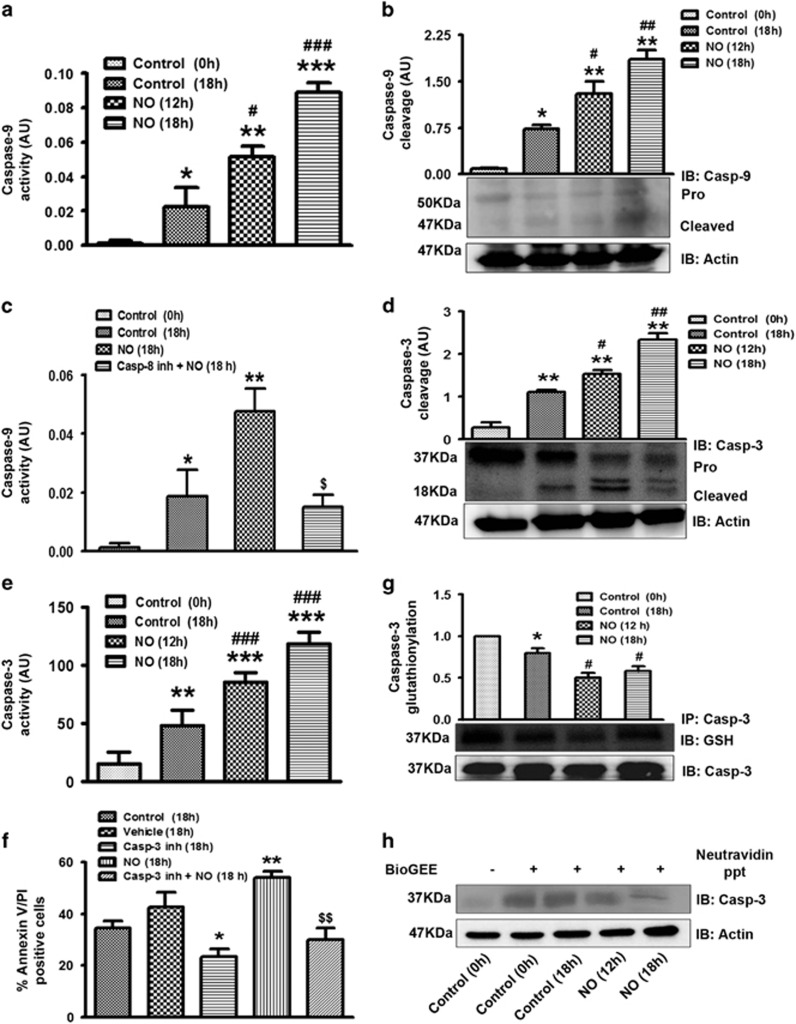
Mitochondrial death pathway in NO-treated cells. (**a**) Caspase-9 activity was monitored in NO-treated human PMNs. Neutrophils (5 × 10^5^cells/ml) were treated with DETA-NO (300 *μ*M) and incubated *in vitro* for 12 and 18 h in a CO_2_ incubator. Cells were then lysed with caspase lysis buffer and incubated with caspase-9 specific chromogenic substrate. Absorbance was monitored at 495 nm (*N*=5). (**b**) Caspase-9 cleavage as measured by western blotting (*N*=3). (**c**) Caspase-9 activity in NO pre-treated cells in presence of caspase-8 inhibitor (casp-8 inh*; N*=5). (**d**) Pro and cleaved caspase-3 as detected by western blotting using caspase-3 antibody (*N*=5). (**e**) Caspase-3 activity was measured in control and NO-treated human PMNs after 12 and 18 h of incubation using caspase-3 specific substrate acetyl-Asp-Glu-Val-Asp-7-amino-4 methylcoumarin (150 *μ*M) (*N*=6). (**f**) NO-induced apoptosis was measured in presence of caspase-3 inhibitor (Casp-3 inh) by Annexin V-PI labelling (*N*=3). (**g**) Caspase-3 was immunoprecipitated from NO-treated neutrophil lysate by anti-pro-caspase-3 antibody and blotted with anti-GSH antibody. (**h**) Treatment of the cells with BioGEE and glutathionylated proteins were precipitated with neutravidin beads. Caspase-3 was identified by western blot using anti-pro-caspase-3 antibody (*N*=3). Data are shown as the mean±S.E.M. of three to five independent experiments. *P<0.05, **P<0.01, ***P<0.001 *versus* control neutrophils, ^#^*P*<0.05, ^##^*P*<0.01, ^###^*P*<0.001 *versus* 18 h incubated apoptotic neutrophils, ^$^*P*<0.05, ^$$^*P*<0.01 *versus* NO-treated neutrophils

tBID translocation to mitochondria leads to caspase-9 activation and commencement of mitochondrial death pathway. Consistent with mitochondrial membrane depolarisation, caspase-9 activity was also enhanced in NO-treated cells (Figure 3a). Caspase-9 cleavage in NO-treated cells was monitored and was found to be enhanced at 12 and 18 h of *in vitro* culture (Figure 3b). In presence of caspase-9 inhibitor, NO-induced apoptosis was found to be less as compared with control cells (Supplementary Figure S1C). However, Caspase-9 glutathionylation signal was not affected in NO pre-treated cells (Supplementary Figure S1D). To confirm caspase-8 mediated caspase-9 activation, NO-induced apoptosis and caspase-9 activation were monitored both in caspase-8 inhibitor pre-treated and NO stimulated cells. Caspase-9 activity was reduced in presence of caspase-8 inhibitor (Figure 3c). We next studied the role of caspase-3 in NO-induced apoptosis where Caspase-3 cleavage and its activity were found to be significantly enhanced in cells pre-treated with NO (Figures 3d and e). Furthermore, NO mediated increased apoptosis was also attenuated by caspase-3 inhibitor (Figure 3f). Caspase-3 deglutathionylation was also augmented in NO pre-treated cells (Figure 3g). BioGEE labelling of the glutathionylated proteins further confirmed caspase-3 deglutathionylation in presence of NO (Figure 3h). Since caspase-3 and -9 also undergo nitrosylation, their nitrosylation was monitored using anti-SNO antibody (Supplementary Figures S1E and F).

### Effect of exogenous NO on the apoptosis of mice BMDN

**Figure 4:**
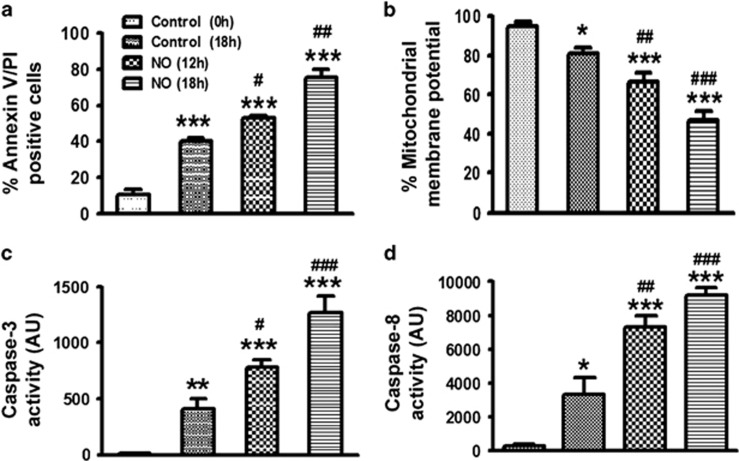
Effect of NO on BMDN. (**a**) Cell apoptosis was monitored in mice bone marrow derived neutrophils. Cells were treated with DETA-NO (300 *μ*M) followed by *in vitro* incubation for 12 or 18 h (*N*=3). (**b**) Mitochondrial membrane potential as assessed by JC-1 labelling (*N*=3). Caspase-3 (**c**) and caspase-8 (**d**) activity was measured in mice BMDN using caspase-3 and caspase-8 specific fluorogenic substrates (*N*=3). Data are shown as the mean±S.E.M. of three independent experiments. *P<0.05, **P<0.01, ***P<0.001 *versus* control neutrophils, ^#^*P*<0.05, ^##^*P*<0.01, ^###^*P*<0.001 *versus* 18 h cultured neutrophils

Similar to our results obtained in human neutrophils, enhanced apoptosis was also observed in NO pre-treated mice BMDN as compared with control mice (Figure 4a). Similarly, mitochondrial membrane potential was also reduced significantly in NO-treated cells (Figure 4b) and ROS generation (Supplementary Figure S1G) was enhanced. Caspase-3 and -8 activities were significantly enhanced in a time-dependent manner during constitutive apoptosis in mice BMDN and in NO-treated cells as compared with the vehicle-treated cells (Figures 4c and d).

### Extrinsic and intrinsic pathways in NCF-1 KO mice

**Figure 5:**
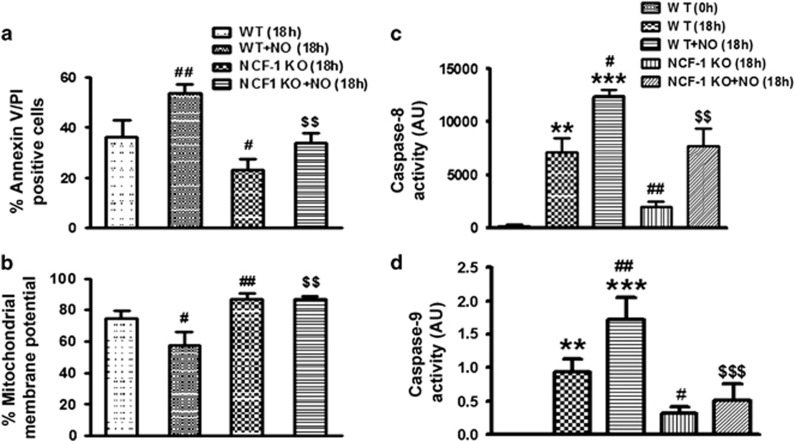
Extrinsic and intrinsic pathways in NCF-1 KO mice. (**a**) Apoptosis in control and NCF-1 KO mice BMDN (*N*=3). (**b**) Mitochondrial membrane potential monitored by JC-1 labelling in WT and NCF-1 KO BMDN after *in vitro* incubation for 18 h (*N*=3). Caspase-8 (**c**) and caspase-9 (**d**) activity in WT and NCF-1 KO mice BMDN following treatment with vehicle or DETA-NO (300 *μ*M; *N*=3). Data are shown as the mean±S.E.M. of three independent experiments. **P<0.01, ***P<0.001 *versus* control neutrophils, ^$$^*P*<0.01, ^$$$^*P*<0.001 *versus* NO-treated neutrophils, ^#^*P*<0.05, ^##^*P*<0.01 *versus* 18 h cultured neutrophils

Our results mark the importance of NO in apoptosis of neutrophils via ROS accumulation and subsequent activation of caspase-8, and mitochondrial death pathway which were then confirmed using NCF-1 KO BMDN. Apoptosis and mitochondrial membrane potential were monitored in wild type (WT) and NCF-1 KO BMDN after 18 h of *in vitro* cell culture. Less apoptosis was observed in NCF-1 KO BMDN as compared with the WT mice. Importantly NO-induced apoptosis was also reduced in NCF-1 KO mice BMDN as compared with the WT BMDN (Figure 5a). NO-induced reduction in mitochondrial membrane potential (Figure 5b) was prevented in NCF-1 KO mice BMDN demonstrating role of NOX in NO-induced apoptosis. Furthermore, NO mediated activation of caspase-3, 8 and 9 was also less in NCF-1 KO mice. There was significant reduction in the activity of caspase-3 (Supplementary Figure S1H), caspase-8 (Figure 5c) and caspase-9 (Figure 5d) in BMDN from NCF-1 KO mice as compared with the BMDN from WT mice after NO treatment. NO-induced BID cleavage was also reduced in BMDN from NCF-1 KO mice (Supplementary Figure S2A) supporting the involvement of NOX mediated ROS generation and BID cleavage in NO-induced apoptosis.

### Status of NO generation and iNOS expression during overnight culture of human and mice PMNs

**Figure 6:**
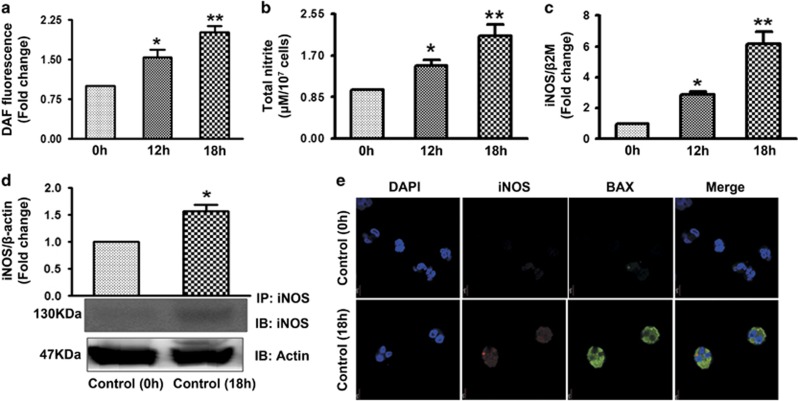
iNOS/NO production during human PMNs spontaneous apoptosis. (**a**) NO generation as measured by DAF. Control human PMNs (1 × 10^6^ cells/ml) were incubated with DAF after 12 and 18 h (*N*=6). (**b**) Total nitrite content measured using Griess reagent in fresh control and apoptotic cell after 12 and 18 h (*N*=6). iNOS expression was monitored by real-time PCR (**c**) in fresh control and apoptotic human PMNs and western blotting (**d**) (*N*=5). (**e**) Immunolabelling of iNOS in fresh and apoptotic neutrophils. Cells were fixed and permeabilized followed by labelling with iNOS and Bax (*N*=2). Scale bar-5 *μ*m. Data represent mean±S.E.M. of three to five independent experiments. *P<0.05, **P<0.01 *versus* control neutrophils

Results obtained so far confirm that exogenous NO-induced neutrophil apoptosis leading us to study next the involvement of endogenous NO in neutrophil spontaneous apoptosis. Overnight cultured human neutrophils (apoptotic; control 18 h) as compared with the freshly isolated cells (Fresh; control 0 h) exhibit more apoptosis (~60% at 18 h), enhanced NO generation (Figure 6a) and accumulation of nitrite content (Figure 6b) in a time-dependent manner. To further assess the source of enhanced NO and nitrite content, the expression of NOS was monitored. iNOS expression was assessed by real-time PCR (Figure 6c) and western blotting (Figure 6d). Expression of iNOS was augmented while expression of nNOS remained almost same (Supplementary Figure S2B). The above mentioned parameters were also monitored in the PMNs obtained from mice bone marrow. In accordance with the above findings enhanced NO generation (Supplementary Figure S2C), nitrite content (Supplementary Figure S2D) and iNOS mRNA (Supplementary Figure S2E) and protein (Supplementary Figure S2F) expression were observed in BMDN during their spontaneous apoptosis in a time-dependent manner. As 55–60% of the cells were apoptotic, the question was to identify whether NO generation and iNOS expression are enhanced in apoptotic or surviving cells. Immunolabelling with Annexin V-FITC/iNOS-PE or Bax/iNOS demonstrated that iNOS expression (Figure 6e and Supplementary Figure S3A) was much more in apoptotic PMNs. Thus NO production and iNOS expression were enhanced in those cells, which were undergoing apoptosis.

### Role of intracellular NOS in spontaneous apoptosis of human and mice neutrophils

**Figure 7:**
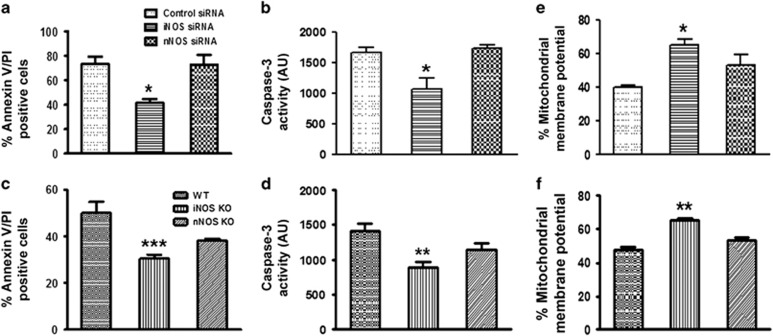
Role of iNOS in neutrophil apoptosis. (**a**) Neutrophil apoptosis was measured by Annexin V-PI labelling in scrambled, iNOS and nNOS silenced human PMNs after 12 h of incubation (*N*=5). (**b**) Caspase-3 activity assay was performed in scrambled, iNOS and nNOS silenced human PMNs (*N*=3). (**c**) Annexin V-PI labelling of BMDN from WT, iNOS and nNOS KO mice after 18 h of *in vitro* culture (*N*=5). (**d**) Caspase-3 activity was monitored in BMDN from WT, iNOS and nNOS KO mice using caspase-3 specific fluorogenic substrate acetyl-Asp-Glu-Val-Asp-7-amino-4 methylcoumarin (150 *μ*M; *N*=5). Mitochondrial membrane potential in control and iNOS silenced human PMNs (**e**) and WT and iNOS KO BMDN (**f**) (*N*=5).Data represent mean±S.E.M. of three to five independent experiments. *P<0.05, **P<0.01, ***P<0.001 *versus* control neutrophils

To further validate the importance of intracellular NO in the apoptosis of PMNs, spontaneous apoptosis was monitored in PMNs after iNOS or nNOS silencing. Apoptosis was significantly reduced in PMNs following iNOS silencing as compared with the control cells transfected with scrambled siRNA, or nNOS silenced cells, suggesting importance of iNOS in the spontaneous apoptosis of human neutrophils (Figure 7a). Pro-apoptotic effect of iNOS was also confirmed by measuring caspase-3 activity. Expectedly caspase-3 activity was also reduced in neutrophils after iNOS silencing during 12 h of culture as compared with the scrambled siRNA transfected control cells, no difference was observed in the caspase-3 activity of PMNs from nNOS silenced cells (Figure 7b). To validate the role of iNOS in PMNs apoptosis, BMDN from iNOS KO, nNOS KO and WT mice were also used. Constitutive neutrophil apoptosis was significantly reduced in BMDN from iNOS KO mice as compared with the WT and nNOS KO mice (Figure 7c). Caspase-3 activity was also less in the bone marrow derived neutrophils from iNOS KO mice after 12 h of incubation as compared with the WT and nNOS KO mice (Figure 7d). In accordance with the previous findings caspase-8 (Supplementary Figure S3B) and 9 activities (Supplementary Figure S3C) were also comparatively lesser in iNOS KO mice BMDN as compared with the WT. Moreover, mitochondrial membrane potential was also less affected after 12 h of incubation *in vitro*, in iNOS silenced human PMNs as well as in BMDN from iNOS KO mice (Figures 7e and f). iNOS inhibitor 1400 W significantly reduced human/mice neutrophil apoptosis at 18 h (Supplementary Figures S3D and E). These results were further confirmed by intraperitoneal administration of LPS in WT and iNOS KO mice (data not shown). LY6G labelling has shown enhanced neutrophil migration in iNOS KO mice which is in accordance with the literature.^18^ In addition, more apoptotic cells (~50%) were observed in WT mice after 8 h of LPS (4 mg/kg) injection as compared with iNOS KO mice (~29%).

## Discussion

Neutrophils, the most abundant leucocytes, have a short life as they undergo spontaneous apoptosis. Although delayed neutrophil apoptosis is required for the clearance of pathogens, prolonged survival is often associated with inflammatory conditions.^19, 20^ During inflammation, PMNs are exposed to various factors such as cytokines, immune modulators and microbial agents, which affect PMNs survival by different mechanisms.^21^ Neutrophil apoptosis by stress-induced signalling is more defined than the understanding of spontaneous apoptosis.^22, 23^ Role of NADPH oxidase in the augmentation of neutrophil apoptosis was confirmed in PMNs from CGD patients and by using NADPH oxidase inhibitor; diphenyleneiodonium which showed reduced neutrophil cell death.^24, 25^ Although NOX have been implicated in neutrophil cell death process, the underlying molecular mechanism is not fully elucidated. In PMNs, ROS generation can also be regulated by NO production. Studies from this lab and others have shown involvement of NO in neutrophil ROS generation.^26, 27^ Furthermore, NO exhibits a biphasic effect on the apoptosis of HL-60 cells (myeloid cell line)^11^ implying importance of NO in neutrophil cell death. Peroxynitrite produced from NO and superoxide also enhanced cell apoptosis.^28, 29^ Paracrine action of NO released from endothelial cells is well demonstrated and is associated with delayed neutrophil apoptosis during inflammatory conditions,^30^ whereas the autocrine action of NO being synthesized and released from human PMNs is the least explored. Present study highlights this key regulatory role of NO/iNOS in neutrophil apoptosis.

Significance of NO/iNOS in neutrophil apoptosis was explored by using NO donor, silencing of iNOS/nNOS as well as by using BMDN from NCF-1, iNOS and nNOS KO mice. DETA-NO, a NO donor, at 300 *μ*M concentration releases ~0.5–1.5 *μ*M of NO which is closer to the reported pathophysiological levels of NO.^31, 32^ Enhanced neutrophil apoptosis was observed after exogenous treatment with NO (DETA-NO) in a time-dependent manner with most significant effect at 12 and 18 h of NO treatment. These sub-optimal time points were used for further study because majority of the neutrophils became apoptotic after 18 h of NO treatment. Previous studies from this lab have shown that NO (DETA-NO; 100 *μ*M) treatment for 3 h promotes NETs release without inducing apoptosis of adhered PMNs,^33^ whereas non-adherent PMNs as used in the present study exhibited augmented apoptosis following long-term exposure of NO (12 to 18 h); yet in both cases the addition of NO led to enhanced ROS generation. Could it be possible that NO-induced apoptosis or NETosis depends on the duration of the exposure as well as the physical state of cells. Cells in suspension represent PMNs as in flowing blood^34^ while adherent cells are closer to the cells found at inflammatory site.^35^ The question, how does NO modulate apoptosis in cell suspension and NETosis in adhered cells, needs further experimentation and is currently under investigation in our lab.

To further delineate the involvement of caspases, their cleavage and activation were monitored in NO-mediated neutrophil apoptosis. During neutrophil apoptosis, ROS accumulation led to enhanced cleavage and caspase-8 activation.^5^ Instead of conventional Fas mediated commencement, ROS involved a novel mechanism of caspase-8 activation. Scheel-Toellner *et al.* have demonstrated that death receptor blocking by antibody did not inhibit neutrophil apoptosis which underscore Fas-independent caspase-8 activation.^5^ Enhanced caspase-8 cleavage and activation were observed in NO pre-treated neutrophils. Moreover, pharmacological inhibition of NOX with VAS-2870 reduced NO-induced neutrophil apoptosis as well as caspase-8 activation (Figure 2). Activity of all caspases is modulated by various regulatory mechanisms and protein S-glutathionylation is one of them.^16, 17^ As apoptosis involved alteration of redox status^36^ and previous studies have shown role of caspase-3 deglutathionylation in the modulation of cell apoptosis, thus glutathionylation of all caspases was monitored in control and NO-treated apoptotic cells. Glutathionylation of pro-caspase-3 inhibited proteolytic caspase-3 activation^16^ and caspase-9, whereas deglutathionylation of pro-caspase-3 by glutaredoxin induced apoptosis in endothelial cells.^17^ Interestingly, we observed caspase-8 deglutathionylation after NO treatment suggesting critical role of deglutathionylation in the regulation of caspase-8 activity. Involvement of active-site Cys360 cysteine^37, 38^ in the modulation of caspase-8 activity via glutathionylation still remains to be confirmed. Although studies have shown enhanced levels of PSSG and glutathionylation of other proteins in apoptotic cells,^39^ our current observations demonstrate deglutathionylation of caspase-8 during apoptosis. These findings suggest that redox-based protein S-glutathionylation has a high degree of specificity and consequently has the ability to regulate selective pathways. Pro-caspase-8 possesses some weak proteolytic activity and cleaves one another when brought together. Caspase-8 self-cleavage and activation are controlled by oligomerization state of pro-caspase-8.^38, 40^ Glutathionylation might be preventing caspase-8 oligomerization and activation in surviving neutrophils. Deglutathionylation of caspase-3 was also found in NO-treated PMNs though there was not much significant difference in the glutathionylation levels of caspase-9. Although previous studies have shown inhibitory effect of NO on caspase-3 and -9 activation due to S-nitrosylation^41, 42^, in the present study we did not find any significant changes in the nitrosylation levels of caspase-3 and -9 (data not shown). Based on the amount of caspase-8 activation, two different pathways have been reported for processing of caspase-8.^43^ In type I pathway, enhanced caspase-8 activation leads to the activation of effector caspases-for instance caspase-3, whereas in type II pathway there is a lower production of active caspase-8. In this situation, caspase-8 employs the mitochondrial death pathway to carry out apoptosis. Glutathionylation thus could be a molecular switch which modulates level of caspase-8 activation during neutrophil spontaneous apoptosis as deglutathionylation reactions did not completely remove GSH molecules from a protein. The transition from the extrinsic to intrinsic pathway is achieved through caspase-8 mediated processing of BID which is a proximal caspase-8 substrate.^44^ Caspase-8 enhanced BID cleavage which translocates to the mitochondria and activate mitochondrial death pathway.^45^ Loss of mitochondrial membrane potential was detected during neutrophil apoptosis suggesting initiation of mitochondrial death pathway. Reduced caspase-9 activity and maintenance of mitochondrial membrane potential were observed in sepsis patients where neutrophil apoptosis is delayed.^19^ Moreover, it is noteworthy that caspase-9 activity was reduced in presence of caspase-8 inhibitor indicating caspase-8-dependent induction of mitochondrial death pathway and caspase-9 activation (Figure 3). However, caspase-8 and 9 inhibitions did not completely prevent NO-induced apoptosis suggesting towards the involvement of other pathways or the inability of inhibitors to completely block the activity of these enzymes.

Our next objective was to study the role of endogenous NO in neutrophil apoptosis. We therefore, measured NO generation, nitrite accumulation and NOS expression in cells after overnight culture. During *in vitro* culture, neutrophils die constantly by apoptosis with ~20% death at 6 h and 80% at 24 h.^46^ In the present study, we found ~40 and 60% of neutrophil apoptosis at 12 and 18 h of incubation respectively which is in accordance with the literature.^46^ Enhanced NO generation, nitrite content and iNOS expression were observed in human and mice neutrophils undergoing apoptosis (Figure 6). Earlier report also suggested enhanced levels of nitrite in spontaneously ageing neutrophils.^47^ To further confirm enhanced iNOS expression in apoptotic cells, immunocytochemical experiments were performed. Confocal imaging showed enhanced iNOS expression in apoptotic cells highlighting induction of iNOS in the cells undergoing apoptosis. Possible role of iNOS in apoptosis was also supported by the ability of 1400 W, an iNOS inhibitor, in reducing spontaneous apoptosis in mice and human neutrophils as was also observed by others.^48, 49^ Spontaneous apoptosis was also less in iNOS KO mice, whereas it was comparable in WT and nNOS KO mice bone marrow neutrophils (Figure 7). Similarly caspase-3, -8 and -9 activities were reduced in iNOS KO mice BMDN. All the evidences thus point towards enhanced activation of caspase-3, 8 and 9 in NO-treated cells indicating involvement of both extrinsic and intrinsic pathways in NO-induced neutrophil apoptosis. Intraperitoneal administration of LPS in mice induced acute inflammation and neutrophil infiltration followed by neutrophil apoptosis.^18, 50^ Reduced apoptotic neutrophils in the peritoneal cavity of iNOS KO mice further validate the role of iNOS in regulation of apoptosis.

Exaggerated neutrophil survival is often associated with many inflammatory and immunological disease conditions.^51, 52^ Enhanced iNOS expression has also been observed under various inflammatory responses but protective role of iNOS in inflammation has also been reported.^53^ NO derived from iNOS helps in the resolution of lung inflammation and there was a marked delay in repair of lung injury in iNOS KO mice.^54^ Shibata *et al.* have shown that endogenously produced NO regulates neutrophil apoptosis and helps in resolution of inflammation during *Staphylococcus aureus* induced inflammation.^18^ Interestingly rodents with more constitutive iNOS have less number of neutrophils (10–25%) while predominance of neutrophils in human blood (70%) is seen along with relatively less iNOS.^55, 56^ Role of NO/iNOS thus should be extensively assessed in the pathological conditions associated with modulation of neutrophil number.

**Figure 8:**
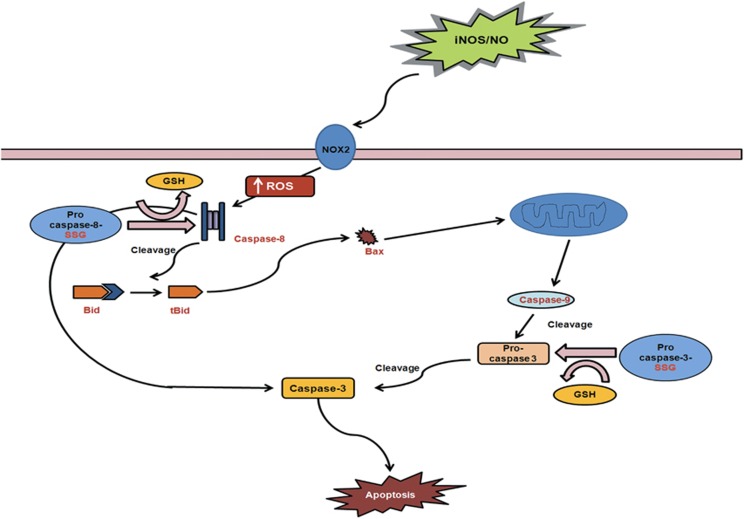
Mechanism of NO/iNOS induced neutrophil apoptosis. NO/iNOS mediated ROS generation induced caspase-8 deglutathionylation, cleavage and activation during neutrophil apoptosis. Active caspase-8 cleaved pro-caspase-3 and caused cell apoptosis. Caspase-8 activation is associated with enhanced cleavage of BID protein which led to initiation of mitochondrial death pathway. Altogether, NO/iNOS plays crucial role in the regulation of neutrophil apoptosis via ROS mediated deglutathionylation and activation of caspase-8, and initiation of mitochondrial death pathway

Summarizing the findings, the present study suggests a pivotal role of NO/iNOS in the regulation of neutrophil apoptosis. NO-induced ROS formation led to caspase-8 deglutathionylation and cleavage followed by BID truncation, caspase-9 activation with subsequent cleavage and activation of caspase-3 (Figure 8). Protection against spontaneous apoptosis in neutrophils from iNOS KO mice further implies role of NO in the regulation of neutrophil survival.

## Materials and Methods

### Materials

Percoll was obtained from Amersham Biosciences Corp. (Uppsala, Sweden). Scrambled, iNOS and nNOS siRNA, anti-iNOS-PE, anti-Bax-PE and CD15-PE antibodies were procured from Santa Cruz Biotechnology (Santa Cruz, CA, USA). Anti-caspase-3, caspase-8, caspase-9, BID and Bax antibodies were purchased from Cell Signalling Technology (Danvers, MA, USA). The Annexin V-PI kit and LY6G-FITC antibody were obtained from BD Biosciences (San Jose, CA, USA). TUNEL Apoptosis detection kit was procured from EMD Millipore (Billerica, MA, USA). Biotinylated GSH monoethyl ester (BioGEE) from Molecular Probes (Eugene, OR, USA) and anti-glutathione monoclonal antibody was purchased from Virogen (Watertown, NY, USA). VAS-2870 was obtained from Enzo Life Sciences (New York, NY, USA). Immunoprecipitation starter pack was procured from GE Healthcare (Piscataway, NJ, USA). All the primers used in the study were obtained from Integrated DNA Technology (India). 1400 W was obtained from Cayman Chemical (Ann Arbor, MI, USA). 2,7-dichlorofluorescein diacetate (DCF), DETA-NONOate (DETA-NO), lipopolysaccharides (from *E. coli*, serotype 0111:B4), caspase-3 substrate (N-Acetyl-Asp-Glu-Val-Asp-7-amido-4-methylcoumarin), caspase-8 substrate (N-acetyl-ile-glu-thr-asp-7-amido-4-methylcoumarin), caspase-9 Substrate, JC-1 (5,5′,6,6′-114 tetrachloro-1,1′,3,3′-tetraethylbenzimidazol carbocyanine iodide), propidium iodide (PI), caspase-3 inhibitor (N-Acetyl-Asp-Glu-Val-Asp-al), caspase-8 inhibitor (Z-Ile-Glu(O-ME)-Thr-Asp(O-Me) fluoromethyl ketone), cell permeable caspase-9 inhibitor and all other chemicals used in the study were obtained from Sigma Aldrich Co. (Saint Louis, MO, USA).

### Isolation of PMNs from human blood and mice bone marrow

Neutrophils were isolated from the blood of the healthy volunteers as described previously.^12, 57^ C57BL/6 control, iNOS, nNOS and NCF-1 KO mice were procured from The Jackson Laboratory (Bar Harbor, ME, USA), and age of the mice used in the study was between 12 and 16 weeks. PMNs from human peripheral blood and mice bone marrow were isolated using Percoll density gradient centrifugation method. Isolation was performed under sterile condition and cells were cultured in CO_2_ incubator to avoid effect of toxins or bacteria. Purity of the isolated PMNs from human and mice was ascertained by CD15 and Ly6G labelling respectively using flow cytometer. The study protocols were approved by the human ethical committee of King George Medical University (KGMU) and institutional human and animal ethics committee of CSIR-CDRI, Lucknow, India.

### Measurement of apoptosis

Apoptosis in human PMNs was measured using Annexin V- PI kit. Control human neutrophils or iNOS/NCF-1 KO mice neutrophils and NO pre-treated cells (5 × 10^6^ cells/ml) were cultured in RPMI-1640 (with 10% FBS) *in vitro* for 12 and 18 h. Cells were then resuspended in Annexin V binding buffer and stained with Annexin V-FITC and PI. In all the experiments, control apoptotic cells used for the comparison were 18 h cultured except for silencing experiments where experiment was terminated after 12 h of incubation. Twenty thousand events were acquired on FACS Calibur (Becton Dickinson, NJ, USA) and analysed by Cell Quest software (Becton Dickinson, NJ, USA).

### LPS induced inflammation and determination of peritoneal neutrophil apoptosis

WT and iNOS KO mice were injected with 4 mg/kg (sublethal dose) of LPS in 500 *μ*l PBS intraperitoneally.^50^ After 8 h of injection, peritoneal exudate cells were collected using ice-cooled PBS. Neutrophil number was monitored by LY6G labelling. Apoptosis was measured by Annexin V-PI labelling.

### iNOS and nNOS silencing

iNOS and nNOS silencing was performed as described previously^57^ with some modifications (using Nucleofactor II electroporation device (Amaxa Biosystems, Cologne, Germany) and programme T-019). Human neutrophils (2 × 10^6^ cells/ml) were resuspended in 100 *μ*l of nucleofector^R^ solution V (Lonza, Cologne, Germany) containing 5 *μ*g of iNOS, nNOS or control siRNA. After silencing, cells were cultured in RPMI-1640 (with 10% FBS and without GM-CSF) for 12 h and cell viability was assessed by Annexin V-PI labelling. Cells were analysed at 12 h, as procedure used for transfection also adversely affects cells with ~95% cell death at 18 h.

### ROS and NO generation

PMNs (1 × 10^6^ cells/ml) treated with DETA-NO (300 *μ*M) were cultured for 12 and 18 h followed by incubation with DCF (10 *μ*M) or DAF (5 *μ*M) for 10 min. Ten thousand events were acquired on FACS Calibur and subsequently ROS and NO generation was analysed using the Cell Quest Programme.

### Immunolabelling of iNOS

iNOS expression was monitored in fresh and apoptotic cells by confocal microscopy. Cells were fixed with 4% (w/v) paraformaldehyde (PFA) for 30 min followed by permeabilization with 0.2% Triton X-100. Cells were then resuspended in 10% (v/v) goat serum (prepared in PBS) for 30 min to prevent non-specific labelling. Cells were then incubated overnight with anti-iNOS (1 : 200) and Bax (1 : 200) antibody at 4 °C followed by staining with Alexa fluor 488 and 647 secondary antibodies at room temperature for 1 h.

For Annexin V and iNOS dual labelling, cells were fixed with PFA and resuspended in Annexin V binding buffer. Cells were then labelled with Annexin V-FITC and iNOS-PE for 1 h.^58^ Nuclei were stained with DAPI (1 *μ*g/ml) for 15 min. Samples were mounted in the mounting medium and images were acquired in confocal microscope (Olympus, Japan).

### Total nitrite estimation

Nitrite content in cell was measured by Griess method.^59^ Cells were pelleted, lysed with hypotonic TKM solution ((25 mM Tris-HCl pH-7.4, 5 mM KCl, 1 mM MgCl_2_ and 1% Nonidet P-40 (NP-40)). After reduction with pre-activated Cadmium pellets for 4 h, supernatant was treated with equal volumes of Griess reagent and kept at 37 °C for 30 min in dark. Absorbance was taken at 545 nm. Concentration of total nitrite (nitrate+nitrite) was calculated using sodium nitrite as standard.

### Caspase activity assay

Caspase activity was measured as described previously.^11^ Cells were lysed with caspase lysis buffer (150 mM Hepes, pH-7.4, 450 mM NaCl, 150 mM KCl, 1.2 mM EGTA, 1.5% NP-40, 0.3% Chaps, 30% sucrose, 10 mM DTT, 3 mM phenyl-methylsulfonyl fluoride (PMSF)) containing caspase-3 specific substrate; acetyl-Asp-Glu-Val-Asp-7-amino-4 methylcoumarin (150 *μ*M) to assess changes in the caspase-3 enzyme activity. Cell lysates were incubated at 37 °C for 30 min. Fluorescence of cleaved 7-amino-4 methylcoumarin was measured at 30 °C using excitation/emission (ex/em) 380 nm/460 nm wavelengths.

Caspase-8 activity was measured in the cell lysates using caspase-8 specific fluorogenic substrate; *N*-acetyl-ile-glu-thr-asp-7-amido-4-methylcoumarin (100 *μ*M) and monitoring fluorescence of the cleaved product at ex/em 380/460 nm. Caspase-9 activity was monitored by incubating cell lysates with 100 *μ*M of caspase-9 specific chromogenic substrate Ac-LEHD-pNa (Ac–Leu–Glu–His–Asp-p-nitroanalide) for 1 h at 37 °C. Absorbance was taken at 495 nm using BioTekmicroplate reader (Heilbronn, Germany). The amount of protein lysate was measured using a BCA kit (Pierce, Rockford, IL, USA).

### Measurement of mitochondrial membrane potential

Mitochondrial membrane potential was measured using JC-1 (5,5',6,6'-tetrachloro-1,1',3,3'-tetra-ethyl-benzimidazocarbo-cyaniniodide), a cationic dye which accumulates in the mitochondria in a membrane potential-dependent manner. Cells were labelled with JC-1 (2.5 *μ*g/ml) for 10 min and 10 000 events were acquired and analysed using FACS Calibur.

### TUNEL assay

TUNEL assay was performed as per manufacturer's instructions (EMD Millipore). Cells were first treated with DETA-NO, followed by fixation with 4% PFA for 15 min. After three washings with PBS, cells were incubated with PBS containing 0.5% Tween-20 and 0.2% BSA for 15 min at room temperature. Cells were then incubated with 50 *μ*l of TdT end-labelling cocktail for 60 min at room temperature. After blocking for 20 min, cells were incubated with 50 *μ*l of avidin-FITC solution and incubated in the dark for 30 min at room temperature followed by three times washing with PBS. Fluorescence of the cells was then monitored by flow cytometry.^60^

### Real-time PCR

mRNA expression of iNOS and nNOS was measured by real-time PCR. Total RNA was isolated and cDNA was synthesized as described previously^57^ using RevertAid H Minus first-strand cDNA synthesis kit (Fermentas Life Science, Vilnius, Lithuania). Primers used in the study have been listed in the Supplementary Table S1.

### Immunoprecipitation, western blot and BioGEE

PMNs were lysed in ice-cold radio immunoprecipitation assay (RIPA) buffer (PBS containing 1 mM EDTA, 1 mM sodium orthovanadate, 1 mM sodium fluoride, 1 *μ*g/ml aprotinin, 100 *μ*g/ml PMSF, 20 *μ*g/ml pepstatin, Protease Inhibitor Cocktail, 5 mM diisopropylfluorophosphate (DFP), 1% Triton X-100 and 0.1% SDS) for 30 min at 4 °C. The supernatant was precleared with protein A/G agarose (Amersham biosciences) and proteins (1000 *μ*g) were immunoprecipitated with 1 *μ*g of iNOS antibody. For monitoring protein S-glutathionylation, cells were lysed with neutrophil lysis buffer containing 0.1 mM EDTA, 0.1 mM EGTA, 1 mM sodium orthovanadate, 1 mM sodium fluoride, protease inhibitor cocktail, 5 mM DFP, 0.5% NP-40 and 100 mM *N*-ethylmaleimide.^57^ After preclearing, protein lysates (1500 *μ*g) were incubated with 1 *μ*g of pro-caspase-3 or -8 antibody overnight at 4 °C. Subsequently, 20 *μ*l of protein A/G agarose was added and incubated for 3 h at room temperature. The beads were washed, resuspended in gel loading buffer (non-reducing), denatured at 95 °C for 10 min and subsequently analysed by western blotting using anti-GSH antibody.

Expression and cleavage of various proteins (BID, caspase-3, caspase-8 and caspase-9) were monitored in the cells (1 × 10^7^ cells/ml) following lysis with RIPA buffer. Protein samples were denatured at 95 °C for 5 min in Laemmli sample buffer, and were subjected to SDS-PAGE. BioGEE labelling of glutathionylated proteins was performed as described previously.^57^ Vehicle and NO-treated cells were labelled with BioGEE 6 h before termination. Proteins were precipitated with neutravidin beads and separated by reducing SDS-PAGE. Caspase-3/8 was identified by western blot using anti-pro-caspase-3 or pro-caspase-8 antibody.

### Statistical analysis

Data have been reported as mean±S.E.M., representing the results from at least 3–5 independent experiments. The data were analysed by one-way ANOVA followed by Newman–Keul's post-analysis. Control and treated samples were compared using Student's *t*-test and *P*-value <0.05 was considered statistically significant.

## Abbreviations

BMDN: bone marrow derived neutrophils
DFP: diisopropylfluorophosphate
ex/em: excitation/emission
iNOS: inducible nitric oxide synthase
KO: knockout
NCF-1: neutrophil cytosolic factor-1
PFA: paraformaldehyde
PMSF: phenyl-methylsulfonyl fluoride
RIPA: radio immunoprecipitation assay
ROS: reactive oxygen species
WT: wild type
