# Supplementary material for: Nitric oxide-mediated apoptosis of neutrophils through caspase-8 and caspase-3-dependent mechanism
Source: Cell Death Dis. 2016 Sep 1;7(9):e2348–. doi: 10.1038/cddis.2016.248 (PMC5059853; doi:10.1038/cddis.2016.248)
Supplement: Supplementary Table S1 [file cddis2016248x1.doc]

**Table 1: List of primer sequences used for PCR analysis in this study**

| **Gene** | **Primer sequence (5’-3’)** | | **Annealing temperature** | |
| --- | --- | --- | --- | --- |
| Human iNOS | F 5’-TGTGCTCTTTGCCTGTATGC-3’  R 5’-TTGCCAAACGTACTGGTCAC-3’ | 55°C | |  |
| Human nNOS | F 5’-TCTAACAGGCTGGCAATGAAG-3’  R 5’-TCTCTAAGGAAGTGATGGTTGAC-3’ | 57°C | |  |
| Human β2-microglobulin | F 5’-TGACTTTGTCACAGCCCAAG3’  R 5’-AGCAAGCAAGCAGAATTTGG-3’ | 57°C | |  |
| Mice iNOS | F 5’-TGCATGGACCAGTATAAGGCAAGC-3’  R 5’-CTCCTGCCCACTGAGTTCGTC-3’ | 59^0^C | |  |
| Mice nNOS | F 5’-ATGCTCAACTACCGGCTCAC-3’ | 59^0^C | |  |
|  | R 5’-GGTCGCTTTGACTCTCTTGG-3’ |  | |  |
| Mice β-Actin | F 5’-CGTTGACATCCGTAAAGACC-3’  R 5’-TGGAGCCACCGATCCACACA-3’ | 59^0^C | |  |
|  |  |  | |  |
