# Supplementary figures and images for: Nitric oxide-mediated apoptosis of neutrophils through caspase-8 and caspase-3-dependent mechanism
Source: Cell Death Dis. 2016 Sep 1;7(9):e2348–. doi: 10.1038/cddis.2016.248 (PMC5059853; doi:10.1038/cddis.2016.248)

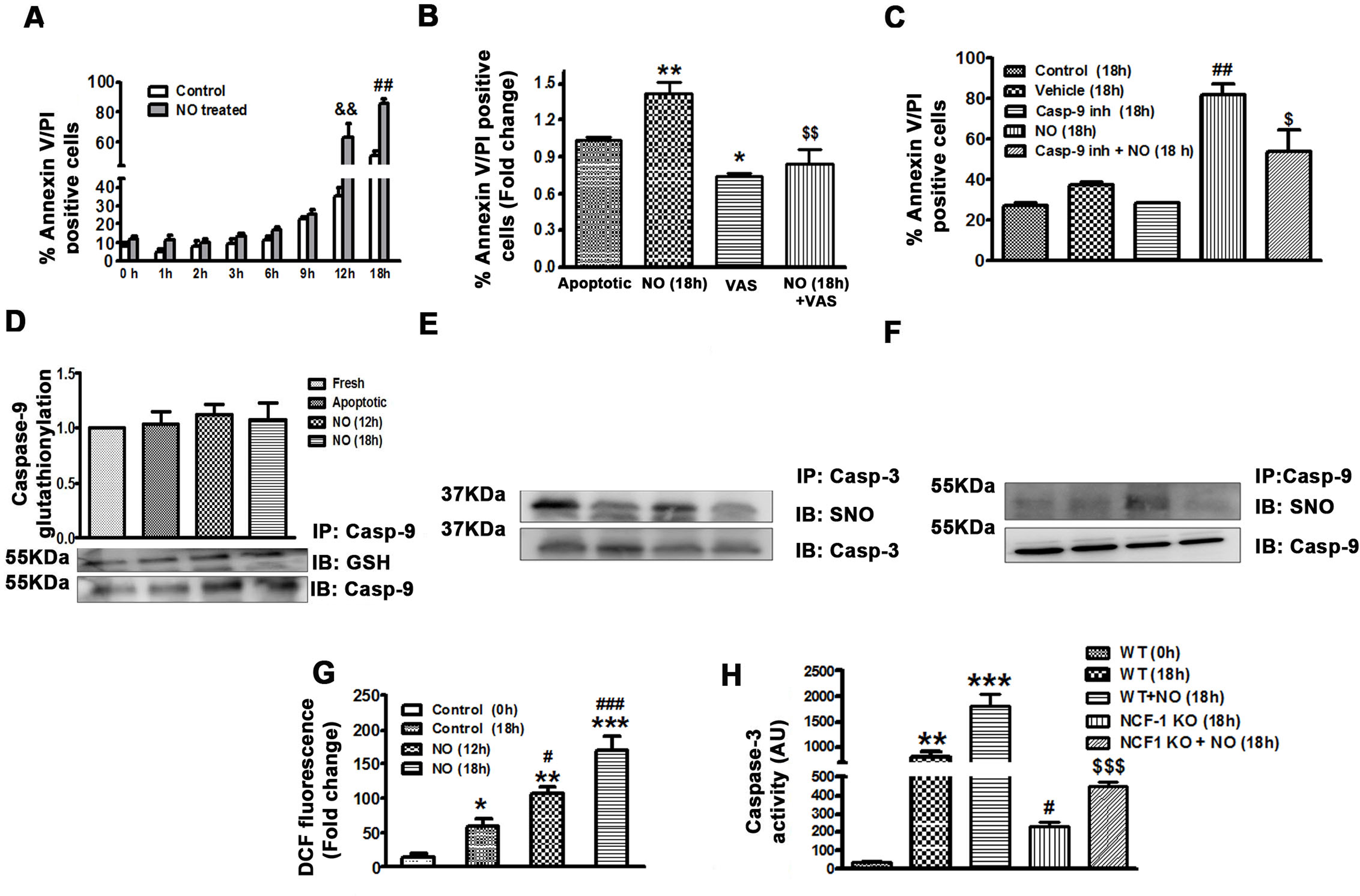

Supplement: Supplementary Figure S1 [file cddis2016248x2.tif]

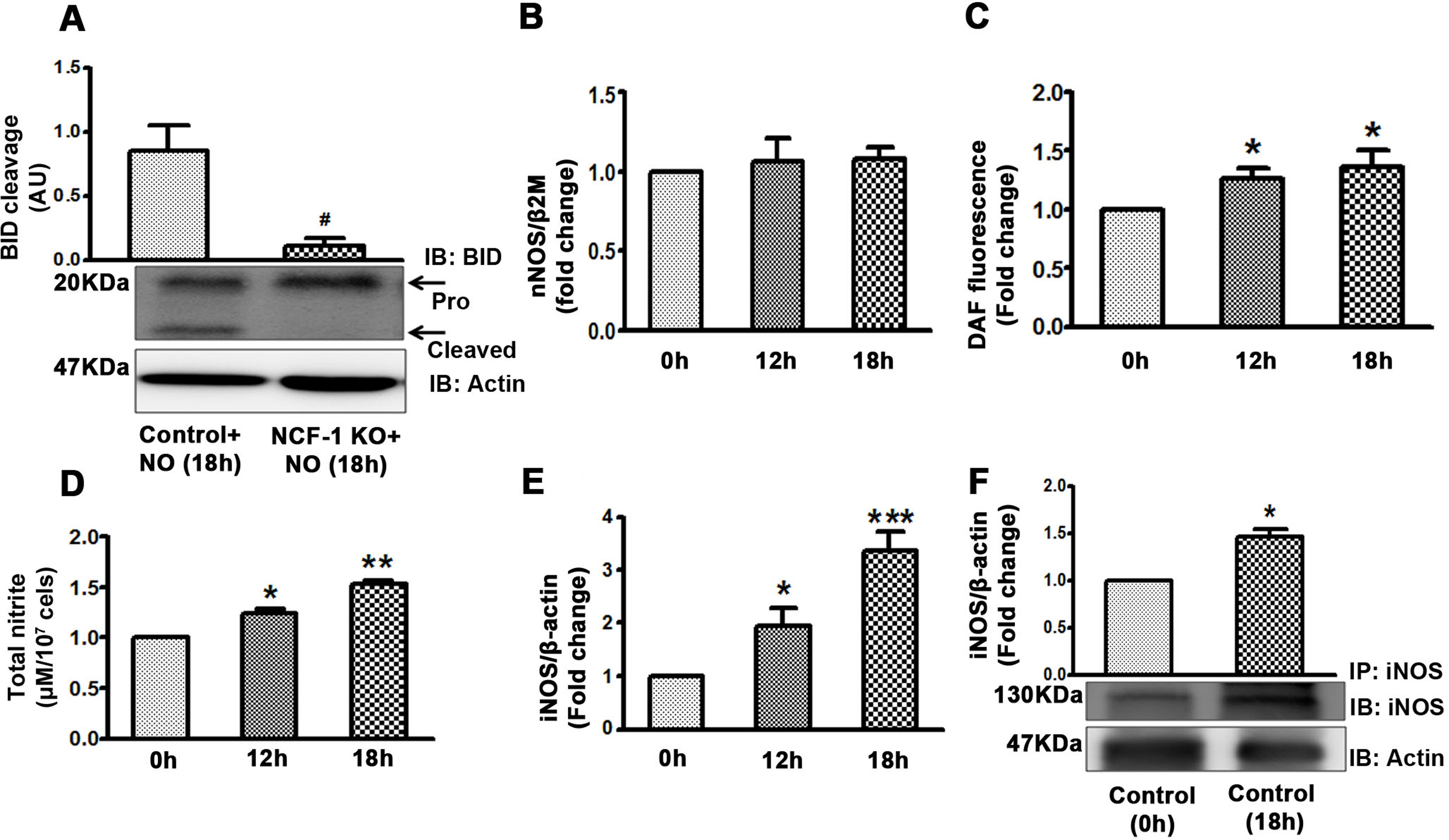

Supplement: Supplementary Figure S2 [file cddis2016248x3.tif]

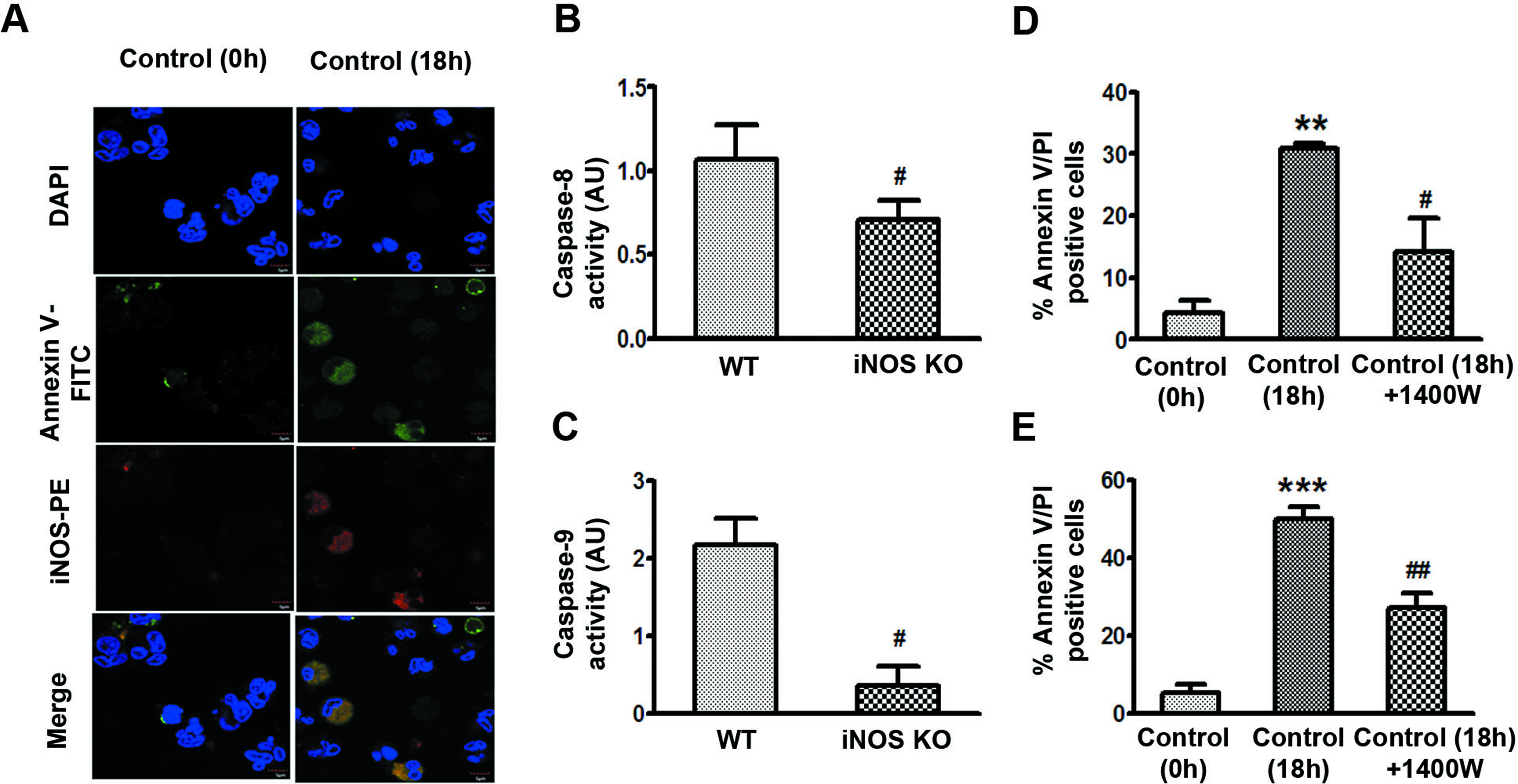

Supplement: Supplementary Figure S3 [file cddis2016248x4.tif]
